# Anthropogenic Disturbance and Climate Change Impacts on the Suitable Habitat of *Sphenomorphus incognitus* in China

**DOI:** 10.1002/ece3.70848

**Published:** 2025-01-18

**Authors:** Kai Chen, Li Ma, Weijun Jiang, Lijin Wang, Li Wei, Hongji Zhang, Ruhao Yang

**Affiliations:** ^1^ College of Ecology Lishui University Lishui Zhejiang China; ^2^ Zhejiang Lishui Ecological Environment Monitoring Center Lishui Zhejiang China

**Keywords:** Biomod2, climate change, human activities, potential distribution, *Sphenomorphus incognitus*

## Abstract

Estimating the impacts of anthropogenic activities and climate change on species' spatial distributions is crucial for conservation. In this study, based on 62 valid occurrence records of *Sphenomorphus incognitus* and 24 environmental factors (19 climate factors, 4 topographic factors, and 1 human activity factor), we utilized the biomod2 combined model platform to predict suitable habitats for *S. incognitus* under two current scenarios (Scenario 1: natural state; Scenario 2: human interference state) and two future climate scenarios (SSP1‐2.6 and SSP5‐8.5) in 2050s and 2090s. The mean true skill statistic (TSS) and the area under the receiver operating characteristic curve (AUC) suggested that the ensemble model yield more precise predictions than those of individual models. Rainfall and slope were identified as the most important factors influencing *S. incognitus* distribution. Human disturbance has significantly reduced suitable habitat by 44.13 × 10^4^ km^2^, which is a decrease in 23.95% compared to natural conditions. Spatial analysis revealed substantial fragmentation of suitable habitat due to human activities. The incorporation of anthropogenic factors into the analysis of future climate scenarios has revealed that the area of suitable habitat exhibits divergent trends. Two distinct scenarios have been identified, each of which results in a reduction in the area of the region by 29.58 × 10^4^ km^2^ and an increase by 27.04 × 10^4^ km^2^, respectively, by the year 2090. The primary influence persists in human activities. The centroid of suitable habitat shifted toward the southeast under SSP1‐2.6 and toward the northwest under SSP5‐8.5. Our findings highlight the significant impact of anthropogenic factors on *S. incognitus* habitat and emphasize the need for conservation measures. Future research should incorporate additional socioeconomic data to further investigate the effects of human disturbance on this species.

## Graphical abstract

We used 62 valid occurrence records of *Sphenomorphus incognitus* and 24 environmental factors (19 climate factors, 4 topographic factors, and 1 human activity factor), and utilized the biomod 2 combined model platform to predict suitable habitats for *S. incognitus* under two current scenarios (Scenario 1: natural state; Scenario 2: human interference state) and two future climate scenarios (SSP1‐2.6 and SSP5‐8.5) in 2050s and 2090s. Our results showed that rainfall and slope were identified as the most important factors influencing *S. incognitus* distribution. Human disturbance has significantly reduced suitable habitat by 44.13 × 104 km^2^, which is a decrease in 23.95% compared with natural conditions. Spatial analysis revealed substantial fragmentation of suitable habitat due to human activities. Our findings highlight the significant impact of anthropogenic factors on *S. incognitus* habitat and emphasize the need for conservation measures.

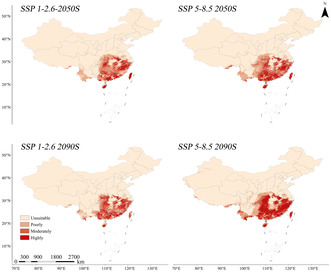

## 1 Introduction

Wildlife is a vital component of biodiversity and plays a crucial role in maintaining the diversity of life in the natural world (Mawdsley, O'malley, and Ojima 2009). Since the 21st century, humanity's ability to reshape nature has progressively strengthened, resulting in substantial degradation of earth's biodiversity (Pereira et al. 2010). Humans encroach on the living space of animals through various activities such as reclaiming farmland (Erb, McShea, and Guralnick 2012), building roads (He et al. 2019), and grazing (Chen and Fu 2000). These activities further affect the range and habitat utilization of animals (Gaynor et al. 2018; Kang 2021). For example, logging activities can affect forest habitats for long periods of time, resulting in animals deliberately avoiding habitats where logged areas are located (Kang 2021). The construction of roads increases the levels of toxic and harmful metals in animal habitats (Zheng et al. 2016), affecting animal food resources to a certain extent (Kang et al. 2020) and indirectly jeopardizing animal health. Due to differences in the intensity and form of anthropogenic disturbances across regions, animals exhibit varying responses and adaptations to human activities (Gaynor et al. 2018; Kang 2021; Zhang et al. 2023). In addition to these direct effects, human‐induced climate warming is also a major contributor to the decline in biodiversity (Hooper et al. 2012; Urban 2015; Shivanna 2022). Studies have shown that over the past 100 years, human activities have caused a global average surface temperature increase of approximately 0.8°C–1.3°C (IPCC 2023). It is projected that by the end of the 21st century, compared with the baseline period, the global average surface temperature will rise by 1.4°C–4.4°C (IPCC 2023). At the same time, climate change has also caused significant shifts in precipitation patterns (Qin et al. 2007). Climate change can threaten the health and even survival of animals by functional disability, metabolic exhaustion, oxidative damage, immunity depression, and accelerated aging (Wang et al. 2024). For example, the extinction risk of species like the Hooded Vulture ( *Necrosyrtes monachus* ) is increasing (Lawer 2024), and the survival of species like the Spiny‐bellied Frog ( *Quasipaa boulengeri* ) is greatly affected by temperature and precipitation (Zhao et al. 2022). Animals can adapt to climate change by altering their physiological tolerance or through migration (Huey and Tewksbury 2009; Lambers 2015). For instance, animals may migrate to higher latitudes or elevations to cope with rising global temperatures (Lambers 2015). Therefore, continuous and effective monitoring of wildlife, along with timely understanding of species dynamics and distribution dynamics, can provide valuable references for wildlife early warning and prevention (Franklin 2010). With current technology, using species distribution models to predict wildlife habitat ranges can provide a foundation for species conservation. This approach has been successfully applied to predict the habitat ranges of species such as the Nilghai ( *Boselaphus tragocamelus* ; Dhami et al. 2023), Giant Panda ( *Ailuropoda melanoleuca* ; Kang et al. 2020), and Red Panda ( *Ailurus fulgens* ; Thapa et al. 2018).

Species distribution modeling (SDM) is a crucial statistical method for studying the correlation between animal or plant and their environment, utilizing species distribution data and environmental data to estimate the ecological niche of a species (Hijmans and Elith 2013). SDMs use different algorithms to estimate the ecological niche of a species, reflecting its habitat preference, probability of occurrence, habitat suitability, or species richness, thus providing an important statistical method to study the correlation between species and their environments (Elith and Leathwick 2009). Biomod2 (Thuiller 2003, 2014; Thuiller et al. 2009, 2020) is an R‐based distribution prediction platform that uses a variety of algorithms, such as generalized linear models (GLM), generalized additive models (GAM), random forest (RF), and maximum entropy models (MaxEnt) to predict the potential distribution of species (Dhami et al. 2023). This platform predicts species and evaluates the results through multiple independent sample splits, ensuring model accuracy. Models that meet the expected accuracy and reliability criteria are selected for further analysis. This approach addresses issues such as data redundancy and overfitting caused by simultaneously using multiple variables, ensuring good fit even with a small sample size, though this may not apply to rare species (Thuiller et al. 2009; Mondanaro et al. 2023).

The skink *Sphenomorphus incognitus*, belonging to the order Squamata, family Scincidae, and genus *Sphenomorphus*, has a snout–vent length of approximately 83–107 mm and a tail length of about 93–182 mm. *S. incognitus* primarily inhabits forested pools and rocky neighborhoods in slow‐moving streams and organic‐rich trails, and is most often found at the edges of forests bordering streams (Fei, Ye, and Jiang 2012; Zhan et al. 2024), so its selective body temperature (29.2°C; Ma, Su, and Ji 2017, unpublished data) is lower than that of other reptile species inhabiting open habitats (Wang et al. 2013; Ma 2018). Currently, most of the studies on the *S. incognitus* focus on reports of new distributions areas and physiological and molecular biological studies. For example, Chen et al. (2017) reported on the new distribution areas of the *S. incognitus* in Zhejiang and Jiangxi provinces. Tang and Huang (2014) reported on its new distribution area in Anhui Province. Ma et al. (2018) studied the reproduction and hatching of *S. incognitus* , while Liang (2019) studied the transcriptome sequencing of the oviductal tissues of the femoral‐scaled lizard and related genes. In light of the expansion of human settlements and intensified human activities, along with climate warming, it is important to examine how small lizards with specialized habitat requirements adapt or respond to these changes. Therefore, this study uses the Biomod2 ensemble model platform to address the following aspects: (1) Predicting the spatial distribution and habitat of *S. incognitus* in current and future climates. (2) To study the spatial distribution of *S. incognitus* under natural and anthropogenic disturbances in the current climate. This will clarify the actual effects of anthropogenic disturbances on the distribution of *S. incognitus* and identify the main environmental factors affecting its distribution. (3) Predicting the spatial distribution pattern of *S. incognitus* under the future climate, and assessing the pattern of change and migration trend of this species in its habitable zone. The study results will provide a scientific reference for the conservation of this small reptile species.

## 2 Materials and Methods

### 2.1 Study Area

Over the past century, global climate warming and habitat fragmentation caused by human activities have had significant adverse effects on species worldwide (IPCC 2023). China, in particular, is highly sensitive to climate change and has experienced warming rates significantly higher than the global average since the mid‐20th century (Jiang, Lv, et al. 2020). According to Zheng, Yin, and Li (2010), China's climate has undergone substantial changes, including increased greenhouse gases, a northward shift of the rainfall zone, and a northward shift of the northern boundaries of the subtropical and warm‐temperate zones. Furthermore, the tertiary climatic zones, such as the mesothermal, warm‐temperate, northern subtropical, and central subtropical zones, have also experienced varying degrees of change. This study focuses primarily on southern China, a region harboring the country's richest biodiversity. It is home to numerous national parks, such as Hainan Tropical Rainforest National Park, Wuyishan National Park, Xishuangbanna National Forest Park, and a multitude of natural reserves. Concurrently, this region is highly developed economically, with intense human activity. Rapid urbanization and extensive infrastructure development exacerbate habitat fragmentation and increase extinction risks for wildlife (Hang et al. 2020; Jia et al. 2024). The combined impact of human activities and climate change poses a significant threat to wildlife survival. Considering the combined effects of human activities and climate change, southern China serves as an ideal setting for biodiversity research.

### 2.2 Data Preparation

#### 2.2.1 Species Occurrence Records

**FIGURE 1:**
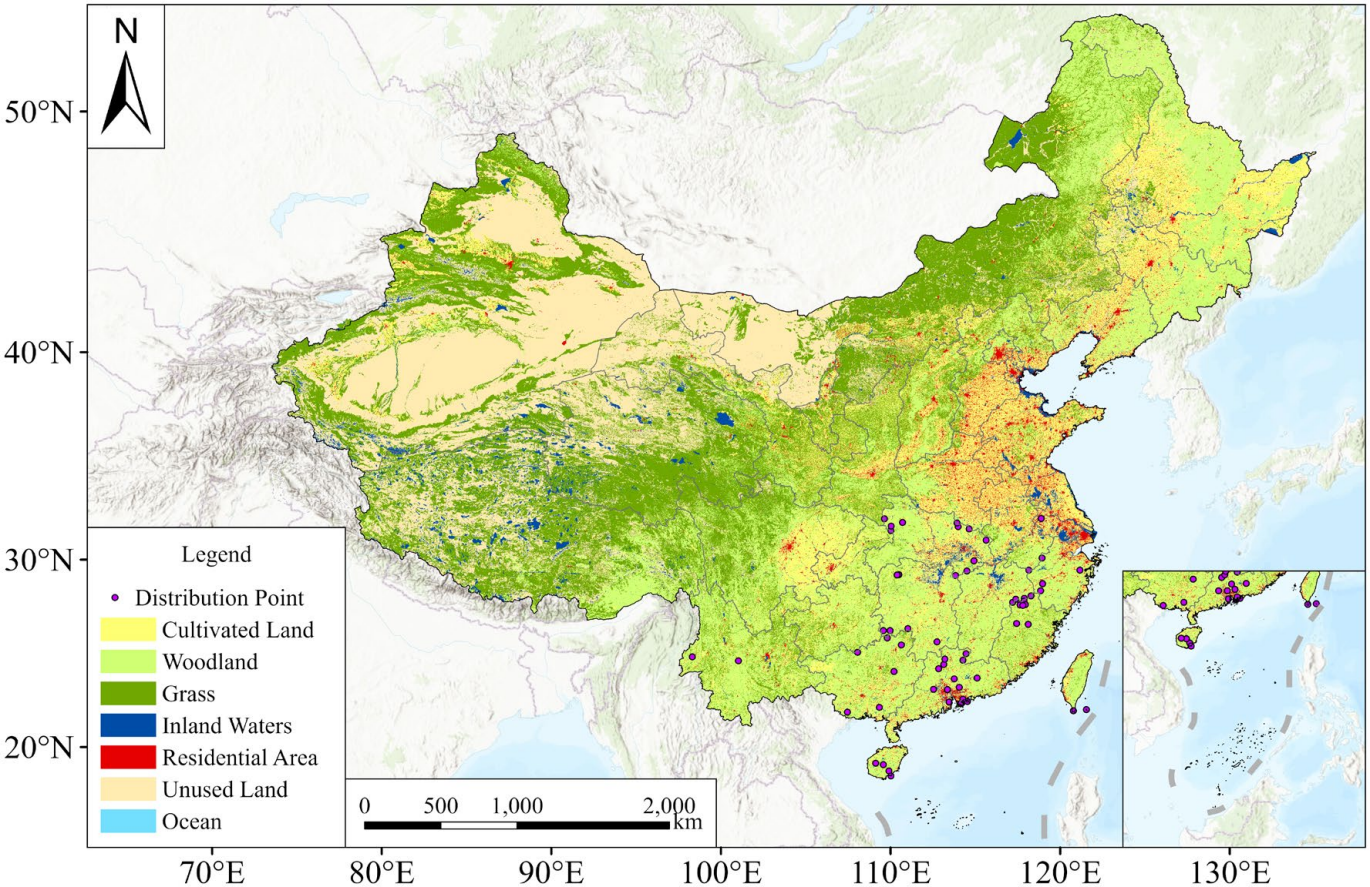
Geographic distribution records of *Sphenomorphus incognitus* in China.

A total of 82 distribution points for *S. incognitus* were obtained from field surveys and literature reports (Table S1). To reduce spatial autocorrelation among the occurrence records, we performed a distance analysis using the Euclidean distance tool in ArcGIS Pro. Points within a 30 km radius were randomly filtered, retaining only one point within each radius. This process resulted in a final dataset of 62 distribution points used for modeling (Figure 1).

#### 2.2.2 Environmental Data

This study selected 23 natural environmental factors and 1 human activity factor (Table 1). The natural environmental factors included 19 climate variables (bio1‐bio19) obtained from the WorldClim version 2.1 database (http://www.worldclim.org/) and 4 terrain variables (elevation, slope, aspect, and hillshade) derived from the GEBCO digital elevation model (DEM) (www.gebco.net). The human disturbance factor was derived from the China's national land use and cover change (CNLUCC) dataset in the resource and environmental science data center of the Chinese academy of sciences (www.resdc.cn). We used two future climate scenarios (SSP1‐2.6 and SSP5‐8.5) from the BCC‐CSM2‐MR climate system model of the National (Beijing) Climate Center in CMIP6, resulting in four combinations: SSP1‐2.6_2050S, SSP1‐2.6_2090S, SSP5‐8.5_2050S, and SSP5‐8.5_2090S.

**TABLE 1:** Environmental variables using in Biomod2.

Datasets	Variables	Description	Units	Participation modeling
Climate	Bio1	Annual mean temperature	°C	
	Bio2	Mean diurnal range (Mean of monthly (max temp–min temp))	°C	Both
	Bio3	Isothermality (Bio2/Bio7) (×100)	—	
	Bio4	Temperature seasonality (standard deviation ×100)	%	
	Bio5	Max temperature of warmest month	°C	
	Bio6	Min temperature of coldest month	°C	
	Bio7	Temperature annual range (Bio5‐Bio6)	°C	Both
	Bio8	Mean temperature of wettest quarter	°C	Both
	Bio9	Mean temperature of driest quarter	°C	
	Bio10	Mean temperature of warmest quarter	°C	
	Bio11	Mean temperature of coldest quarter	°C	
	Bio12	Annual precipitation	mm	Both
	Bio13	Precipitation of wettest month	mm	
	Bio14	Precipitation of driest month	mm	
	Bio15	Precipitation seasonality (Coefficient of variation)	—	Both
	Bio16	Precipitation of wettest quarter	mm	
	Bio17	Precipitation of driest quarter	mm	Both
	Bio18	Precipitation of warmest quarter	mm	
	Bio19	Precipitation of coldest quarter	mm	
Topography	Alt	Elevation	M	Both
	Sl	Slope	°	Both
	SlO	Aspect	—	Both
	Hil	Topographic shadow	—	Both
Human Activities	LUCC	The land use and cover change	—	Scenario 2

All factors were processed using ArcGIS Pro software and map vector files based on the Chinese standard map (review number GS (2023) 2767), These files were obtained from the standard map service website of the State Administration of Surveying, Mapping and Geoinformation (www.mnr.gov.cn). The spatial resolution for all environmental factors was set to 1 km.

**FIGURE 2:**
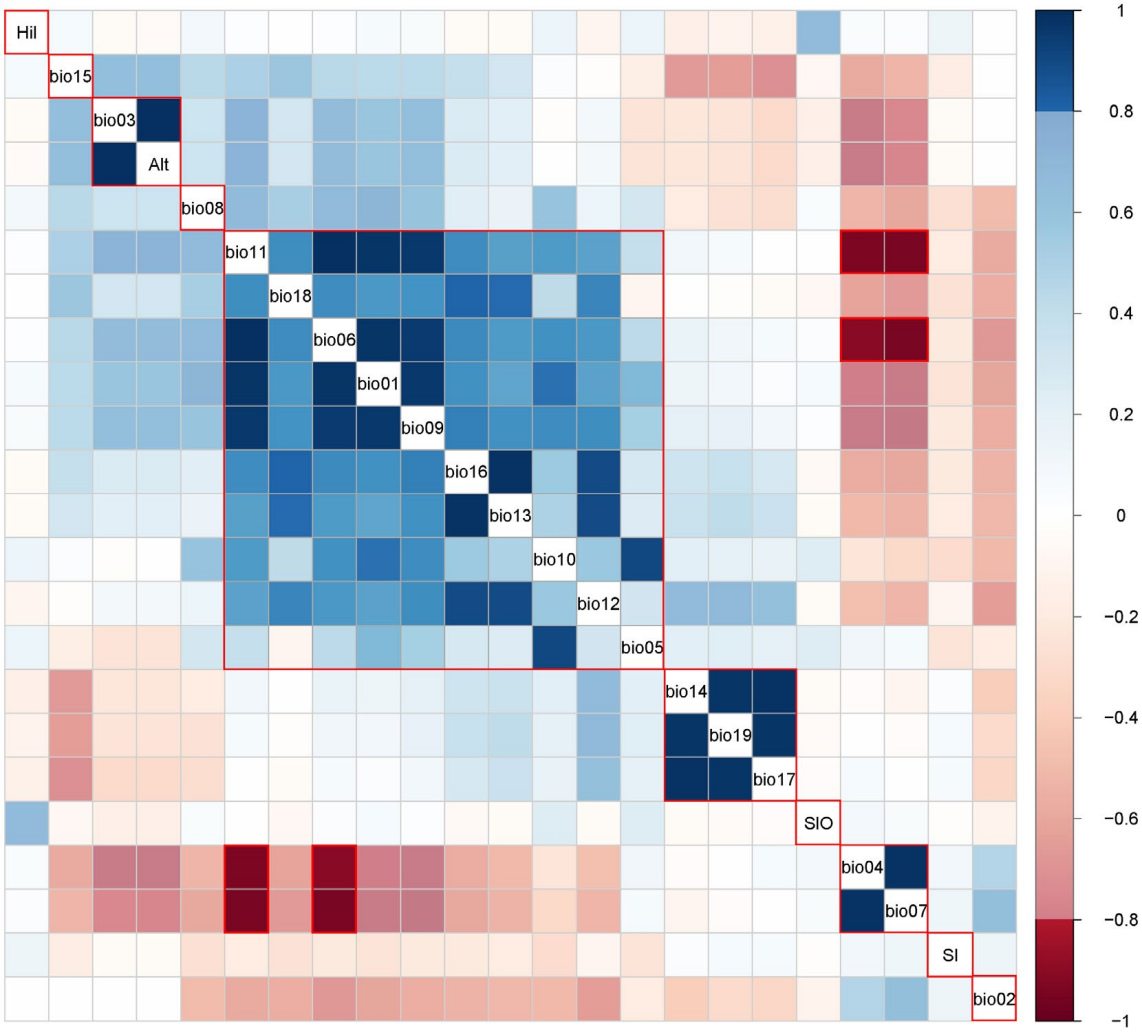
Results of Pearson correlation analysis.

To ensure model effectiveness and avoid overfitting due to high collinearity among natural environmental factors, we conducted a Pearson correlation analysis using SPSS 24 software to identify highly correlated variables. A correlation heatmap (Figure 2) was generated using the Corrplot package in R software version 4.3.1 (Friendly 2002) to visualize the relationships between factors. Based on the analysis results, natural environmental factors with an absolute correlation coefficient |*r*| ≤ 0.8 were selected for subsequent modeling (Li et al. 2023) to ensure that the predictive factors were independent and not highly collinear. The final set of variables included Hil, bio15, Alt, bio08, bio12, bio17, Slo, bio7, Sl, and bio02.

### 2.3 Model Building and Optimization

The modeling process was conducted under two scenarios: Scenario 1 involved using only natural environmental factors, while Scenario 2 included both natural environmental factors and human activity factors. The species distribution data were divided into training (80%) and validation (20%) sets (Allouche, Tsoar, and Kadmon 2006). The Biomod2 platform was used with R software version 4.3.1. Species distribution data and environmental factor data were formatted and loaded into the platform. Within the species' defined range, 500 pseudo‐absence points were generated. All models were run using the default settings of the Biomod2 platform, with 10 repetitions. After completing the runs, the top‐performing individual models were selected for ensemble modeling. Future simulations were conducted using the same approach.

### 2.4 Model Accuracy Validation

The accuracy of the ensemble models was assessed using two evaluation metrics: True Skill Statistics (TSS; Allouche, Tsoar, and Kadmon 2006) and area under the curve (AUC; Fielding and Bell 1997). TSS is used to evaluate the simulation performance of the models, with values closer to 1 indicating better simulation effectiveness. AUC, on the other hand, measures the model's ability to discriminate between presence and absence. An AUC value of 0.5 suggests no discrimination ability, while values between 0.8 and 0.9 indicate good discrimination. AUC values above 0.9 signify the model's ability to accurately simulate the potential distribution of the species (Allouche, Tsoar, and Kadmon 2006; Fawcett 2006).

### 2.5 Random Pairing Experimental Design for Models

To account for the inherent uncertainty in the models and to further validate the impact of environmental variables on species distribution, we conducted a randomness validation experiment in addition to the model accuracy validation. To further validate the impact of environmental variables on species distribution, this study employed two scenario groups for experimentation.

In the main experiment, a threshold of |*r*| ≤ 0.8 was applied for selecting environmental factors. The model was run 10 times to account for variability and reduce the impact of a single run. However, it is possible that the same combination of environmental factors contributed to the reduction in suitable habitats for *S. incognitus* due to human influence. To address this issue, we redesigned the experiment. Specifically, to minimize the effect of identical environmental factor combinations, we applied a threshold of |*r*| ≤ 0.9 to adjust the factor combinations (Li et al. 2020). For the randomness validation experiment, we increased the threshold to |*r*| ≤ 0.9 to reselect the environmental factors. The filtered environmental factors were then modeled using the same method as described above.

In this study, the distribution data of *S. incognitus* were randomly divided into two datasets (A and B) at a 75:25 ratio using the RandBetween function in Excel. Dataset A, comprising 45 distribution sites, was used for model training, while Dataset B, consisting of 17 distribution sites, was reserved for model validation. This division enabled an effective training validation approach, with 75% of the data allocated for training and 25% held out for independent validation. By retaining a substantial portion of the data for validation, this strategy minimized potential experimental variability and ensured that model performance was evaluated on a separate dataset, thereby preserving the integrity of the validation process. Modeling followed the procedures outlined in Section 2.3, “Model Building and Optimization.” To enhance model robustness and reduce variability, the entire modeling‐validation process was repeated ten times.

To analyze the model performance, the model accuracy data were exported to an Excel table using the openxlsx package in R. Due to the increased correlation threshold (|*r*| ≤ 0.9) used in this experiment, two additional indices, the kappa statistic and the critical success index (CSI), were included to account for potential impacts of higher variable correlations on the simulation results. Kappa assesses the agreement between the distributed data and the model simulation results, with values closer to 1 indicating better agreement. CSI, on the other hand, measures the proportion of correct predictions, with values closer to 1 indicating higher correctness (Allouche, Tsoar, and Kadmon 2006; Fawcett 2006).

Therefore, four indicators were used in this experiment for model screening. To avoid the situation of extreme low values of a certain indicator, a conditional selection function was set for the accuracy calculation. The selected accuracy calculation rule is shown in Equation (1), where *K* represents the accuracy information derived from the model package, and the small markers correspond to the names of the specific parameters.

(1) $$\gamma =\frac{1}{4}\times \;\sum \left(\begin{array}{cc} & \text{IF}\left(K_{\mathrm{CSI}}>0.3,K_{\mathrm{CSI}}\times 0.5,K_{\mathrm{CSI}}\times 0.3\right)\\ & +\text{IF}\left(K_{\text{KAPPA}}>0.4,K_{\text{KAPPA}}\times 0.5,K_{\text{KAPPA}}\times 0.3\right)\\ & +\text{IF}\left(K_{\mathrm{ROC}}>0.9,K_{\mathrm{ROC}}\times 0.5,K_{\mathrm{ROC}}\times 0.3\right)\\ & +\text{IF}\left(K_{\mathrm{TSS}}>0.7,K_{\mathrm{TSS}}\times 0.5,K_{\mathrm{TSS}}\times 0.3\right) \end{array}\right)$$

The 20 models with the highest accuracy after each run, including both independent and single models, were numbered and randomly selected using the RandBetween function in Excel. Both the randomly selected models and the random validation set were imported into ArcGIS Pro for visualization and analysis.

Model Randomness Evaluation Method: Scenario 1 served as the control group, and Scenario 2 as the experimental group. If the distribution points of the random validation set appeared in the suitable habitat of the control group but not in the experimental group, the control group scored 1 point. If the distribution points of the random validation set appeared in the suitable habitat of the experimental group but not in the control group, the experimental group scored 1 point. If the distribution points of the random validation set appeared in both the control and experimental groups' suitable habitats, but with different habitat ranks, the group with the higher rank scored 1 point. If the distribution points of the random validation set appeared in both the control and experimental groups' suitable habitats, with the same rank, but there was evident habitat fragmentation within a 50 km radius around the distribution points in the experimental group compared with the control group, the experimental group scored 1 point. If the distribution points of the random validation set appeared in both the control and experimental groups' suitable habitats, with the same rank, and no evident habitat fragmentation, then no points were awarded. This method was used to assess whether human activity factors impact the suitable habitat of the *S. incognitus* .

In this study, the DescTools package in R was used to perform *G*‐test (Hope 1968; Patefield 1981; Agresti 2007; Sokal and Rohlf 2012) to evaluate whether there were statistically significant differences existed in the frequency distribution between the control and experimental groups.

### 2.6 Calculation of Centroid Migration

The Mean Center tool in ArcGIS Pro was employed to calculate the current and future centroids of the suitable habitat. Subsequently, trend maps of centroid migration were generated based on different scenarios.

## 3 Results

### 3.1 Simulation and Accuracy Assessment of Species Distribution Models

**FIGURE 3:**
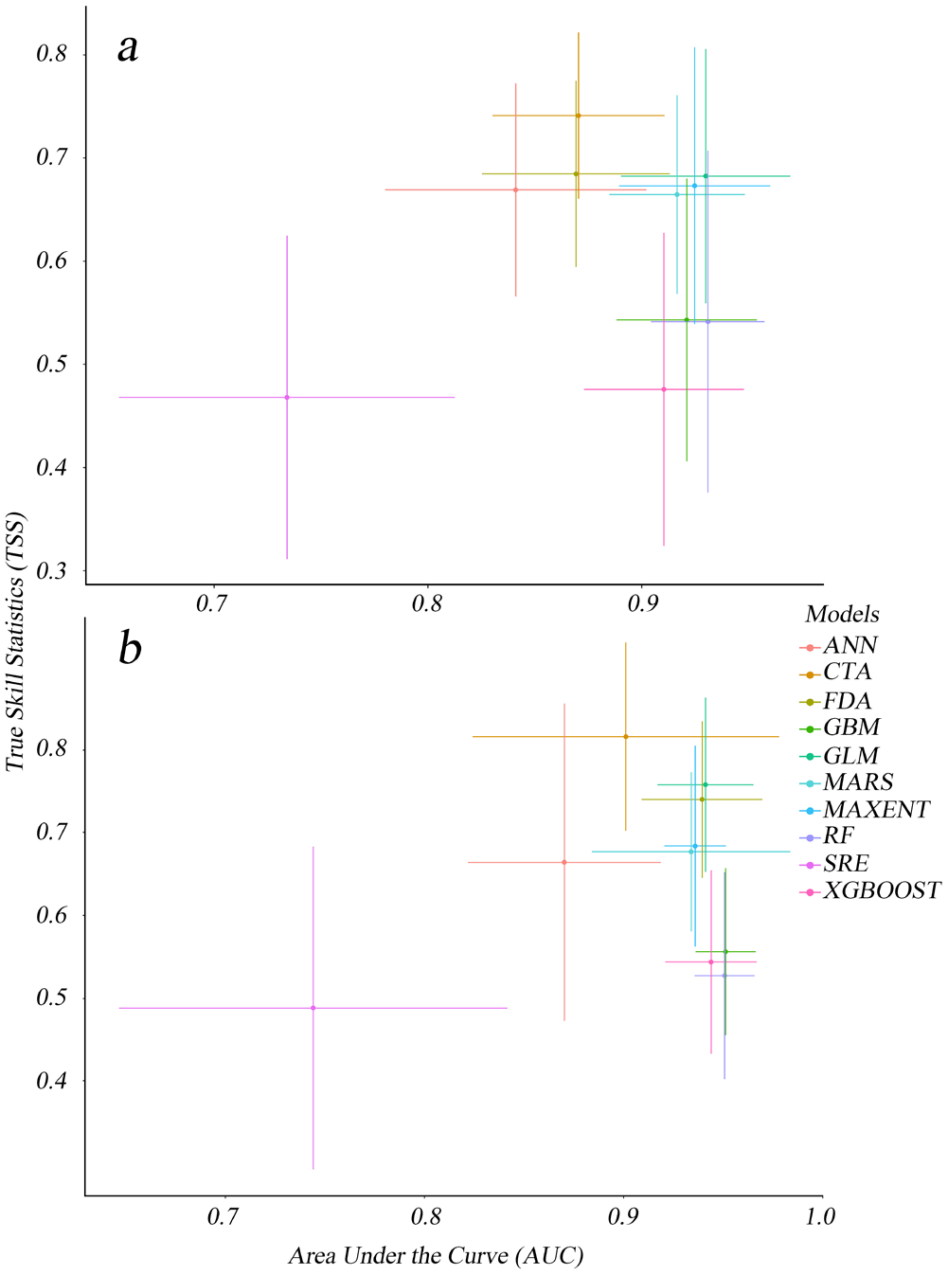
Scatter plots of single‐model performance in two scenarios. (a) Scenario 1; (b) Scenario 2.

After running the distribution data of *S. incognitus* and two sets of environmental variables on the Biomod2 platform for 10 iterations, the ANN models under natural environmental conditions failed to meet performance thresholds 9 times, while under anthropogenic disturbance conditions, the ANN model failed only once. Under the natural environmental scenario (Scenario 1), 91 independent models were generated, while the anthropogenic disturbance scenario (Scenario 2) produced 99 independent models. Accuracy statistics for each scenario were compiled (Figure 3), with the highest TSS values for both scenarios reaching 0.9. The top 5 independent models with the highest TSS and AUC values for each scenario were selected for the model ensemble. Each scenario had 10 models participating in the ensemble, which were integrated into the *EMmodels_TSS* and *EMmodels_AUC* (Table 2). The results showed that the integrated model based on the AUC values outperformed the one based on the TSS values. Additionally, the integrated model demonstrated higher accuracy than the independent models, making the AUC‐based model more reliable.

**TABLE 2:** True skill statistics (TSS) and area under the curve (AUC) for different models in two scenarios.

Scenario		Model	Model repeat order	TSS	AUC
Scenario 1	TSS	CTA	4	0.9	0.95
		ANN	4	0.9	0.95
		GLM	4	0.88	0.991
		MAXNET	4	0.87	0.983
		RF	9	0.847	0.964
	AUC	GLM	4	0.88	0.991
		MAXNET	4	0.87	0.983
		RF	4	0.563	0.98
		GBM	4	0.783	0.977
		GLM	9	0.82	0.968
		EMmodels_TSS		0.903	0.987
		EMmodels_AUC		0.951	0.995
Scenario 2	TSS	CTA	3	0.9	0.95
		CTA	10	0.9	0.95
		CTA	1	0.89	0.945
		GLM	2	0.89	0.953
		CTA	6	0.89	0.945
	AUC	FDA	10	0.857	0.975
		GBM	10	0.627	0.97
		RF	1	0.543	0.968
		GLM	10	0.627	0.967
		GBM	8	0.523	0.966
		EMmodels_TSS		0.879	0.947
		EMmodels_AUC	—	0.929	0.991

### 3.2 Influential Factors Determining Habitat Suitability

**FIGURE 4:**
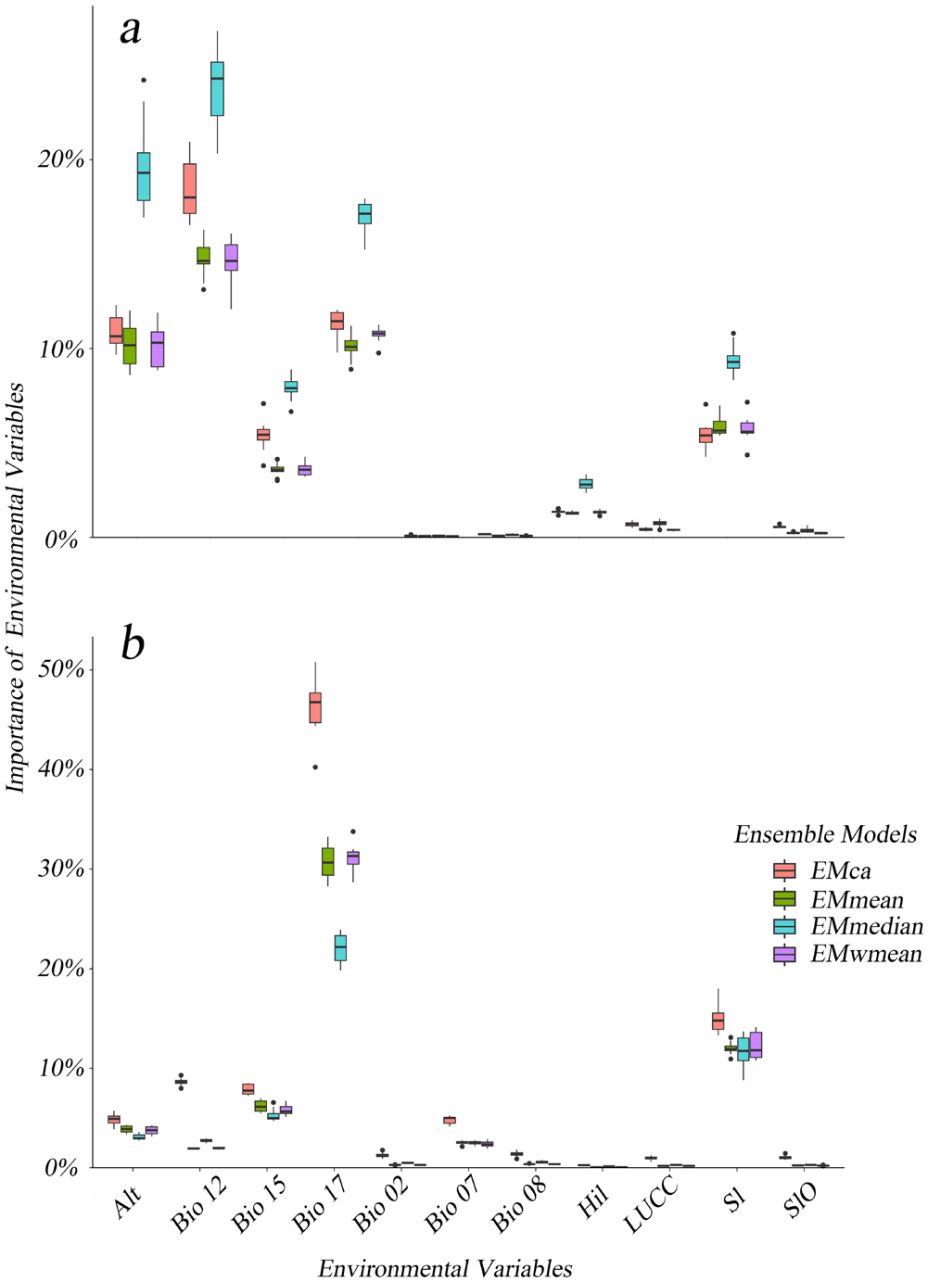
Box plots illustrating the importance of variables in two scenarios. (a) Scenario 1; (b) Scenario 2.

This study established habitat suitability models under two scenarios (Figure 4). The results indicate that the inclusion of anthropogenic factors significantly contributes to habitat fragmentation and reduction. Under natural environmental conditions, the dominant environmental factors are Bio12, Alt, Bio17, Sl, and Bio15, while under anthropogenic disturbance conditions, only Bio17 and Sl remain as the dominant factors. Given that anthropogenic disturbance is inevitable, and both Bio17 and Sl are dominant environmental factors under both natural and anthropogenic disturbance conditions, they are identified as the main factors influencing the changes in the habitat suitability of *S. incognitus* . In this study, anthropogenic factors were the sole experimental variable, showing a very low contribution in the simulation results. However, the experiment included sufficient repetitions to reduce the presence of randomness. To verify whether changes in habitat suitability are due to anthropogenic disturbance factors, random pairing experiments will be conducted.

### 3.3 Current Climatic Suitable Habitat

**FIGURE 5:**
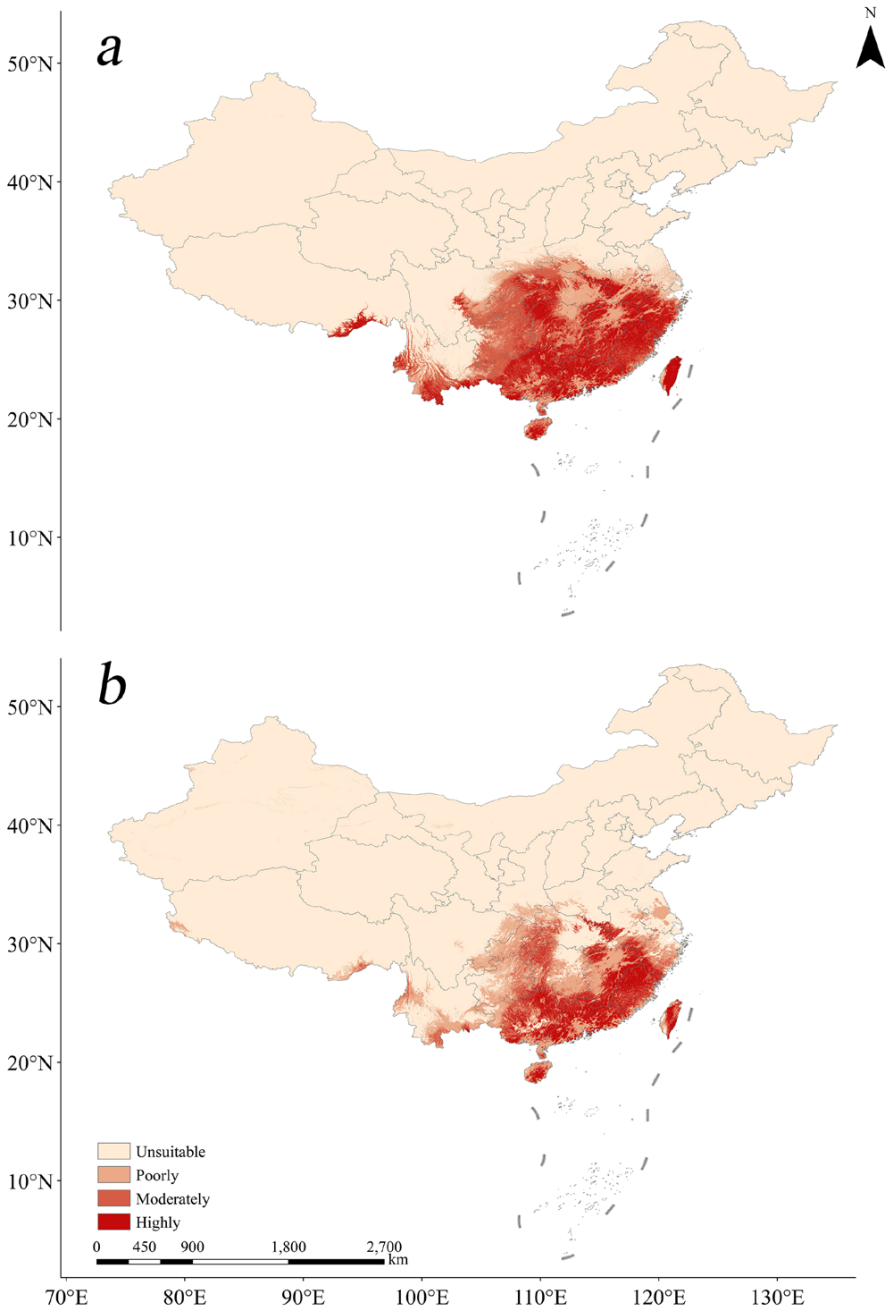
Current models for Scenario 1 (a) and Scenario 2 (b).

Based on the selected optimal ensemble model, the simulated results were classified into four levels using the natural breaks (Jenks) method (Zhu et al. 2018). The four grades were: unsuitable, poorly, moderately, highly (Table 3, Figure 5).

**TABLE 3:** Suitable habitat area (×104 km^2^) of *Sphenomorphus incognitus* in two scenarios under the current climate.

Scenario	Suitable areas	Area (×10^4^ km^2^)	Proportion (%)	Variation (×10^4^ km^2^)
Scenario 1	Highly	61.95	6.47	—
	Moderately	69.94	7.30	—
	Poorly	52.35	5.47	—
	Unsuitable	773.61	80.77	—
Scenario 2	Highly	37.19	3.88	—
	Moderately	30.62	3.20	—
	Poorly	72.3	7.55	—
	Unsuitable	817.74	85.37	—
Contrast (1–2)	Highly	—	—	+24.76
	Moderately	—	—	+39.32
	Poorly	—	—	−19.95
	Unsuitable	—	—	−44.13

A comparison of the current model with Scenarios 1 and 2 was presented below. It is observed that anthropogenic disturbance in Scenario 2 significantly disrupts the continuity of suitable habitat. Furthermore, suitable habitat has been reduced by 44.13 × 10^4^ km^2^, representing a decrease of 23.95%. The data demonstrate that anthropogenic disturbance has a pronounced effect on the suitable habitat.

The results indicate that, under both scenarios, highly suitable areas are concentrated in Hubei, Anhui, Zhejiang, Chongqing, Hunan, Jiangxi, Fujian, Taiwan, Yunnan, Guizhou, Guangxi, Guangdong, Hainan, and southeastern Tibet. However, under the human disturbance scenario, suitable habitat exhibits noticeable fragmentation in regions such as southern Zhejiang, western Chongqing, northern Fujian, southern Yunnan, central Guizhou, and central Guangdong.

### 3.4 Random Pairing Experiment Results and Analysis

The randomized training set and the selected environmental factors were imported into the Biomod2 platform separately, and multiple models were run and repeated 10 times. The value of γ, a metric for a model performance, was calculated for each individual model using Equation (1). The top five independent models with the highest γ values from each group were selected for ensemble modeling. The obtained models were ranked in order of the value of γ calculated by Equation (1) from high to low. The 20 models with the highest γ values were numbered, and random selection was performed using the RandBetween function in Excel. The selection results are presented in Table 4 and Figure S1.

**TABLE 4:** Random pairing experiment model table.

Scenario	Group number	Random serial number	Model (Model repeat order)	CSI	KAPPA	AUC	TSS	γ
Scenario 1	1	16	GLM (1)	0.467	0.597	0.956	0.85	0.359
	2	14	XGBOOST (4)	0.583	0.712	0.971	0.748	0.377
	3	12	ANN (9)	0.421	0.542	0.929	0.789	0.335
	4	9	GBM (2)	0.5	0.632	0.96	0.799	0.361
	5	20	GLM (5)	0.467	0.597	0.966	0.84	0.359
	6	12	ANN (7)	0.444	0.569	0.943	0.799	0.344
	7	10	ANN (5)	0.571	0.698	0.972	0.94	0.398
	8	1	EMmodels (1) (EM)	0.837	0.903	0.993	0.944	0.460
	9	6	GLM (4)	0.471	0.597	0.959	0.88	0.363
	10	20	MAXENT (4)	0.353	0.468	0.917	0.78	0.315
Scenario 2	1	10	MAXENT (4)	0.455	0.596	0.961	0.829	0.355
	2	20	MAXENT (10)	0.4	0.517	0.951	0.81	0.335
	3	18	GLM (3)	0.417	0.554	0.974	0.870	0.352
	4	17	XGBOOST (7)	0.5	0.637	0.972	0.637	0.311
	5	10	GLM (6)	0.389	0.508	0.943	0.749	0.324
	6	9	MARS (1)	0.533	0.662	0.978	0.89	0.383
	7	5	GLM (3)	0.643	0.758	0.994	0.94	0.417
	8	16	FDA (9)	0.471	0.597	0.969	0.809	0.356
	9	5	GBM (10)	0.583	0.712	0.965	0.748	0.376
	10	13	GBM (5)	0.4	0.527	0.958	0.718	0.325

*Note:* Random experimental design for two scenarios in the current climate, including paired group numbers and model rank numbers extracted using Rand Between. The selected models' Area under the curve (AUC) is also listed.

The selected models from Table 4, along with the randomly selected validation sets for both groups, were imported into ArcGIS Pro for visual analysis, resulting in two sets of visual evaluation data. From the table data, it can be observed that the control group scored a total of 34 points in 10 repetitions, while the experimental group scored 74 points, accounting for 21.25% and 46.25% of the total, respectively.

According to the experimental results (Table 5), if only the total scores are considered, the experimental group's predictive accuracy is significantly higher than that of the control group. Analyzing the specific scores reveals that 50 points of the experimental group come from the fragmentation of suitable habitats. As the experimental group incorporates human activity factors, the areas of high‐suitability habitats in the experimental group are mostly lower than those in the control group. However, the experimental group can still accurately predict the randomly selected validation set. Therefore, it is considered that the inclusion of human activity factors enhances the accuracy of the model predictions, enabling a better prediction of the species' suitable habitats.

**TABLE 5:** Results of random pairing experiment table.

Rand group	Control group	Test group	Equality group	Habitat fragmentation score
1	2	8	6	11
2	4	5	7	0
3	2	13	1	10
4	4	10	2	4
5	2	2	12	1
6	4	7	5	5
7	1	9	6	6
8	8	0	8	0
9	2	9	5	3
10	5	11	0	10
Summation	34	74	52	50

*Note:* Includes the experimental data after scoring, along with the compilation of the habitat fragmentation score.

After obtaining the results, *G*‐test was used to assess whether the paired experimental results had a significant difference. The likelihood ratio test results showed a significant difference in the distribution of behavioral frequencies between the two groups (*G* = 26.868, df = 9, *p* < 0.01). Thus, it can be concluded that this paired experimental model had a significant impact.

### 3.5 Future Climate Suitable Habitat

**FIGURE 6:**
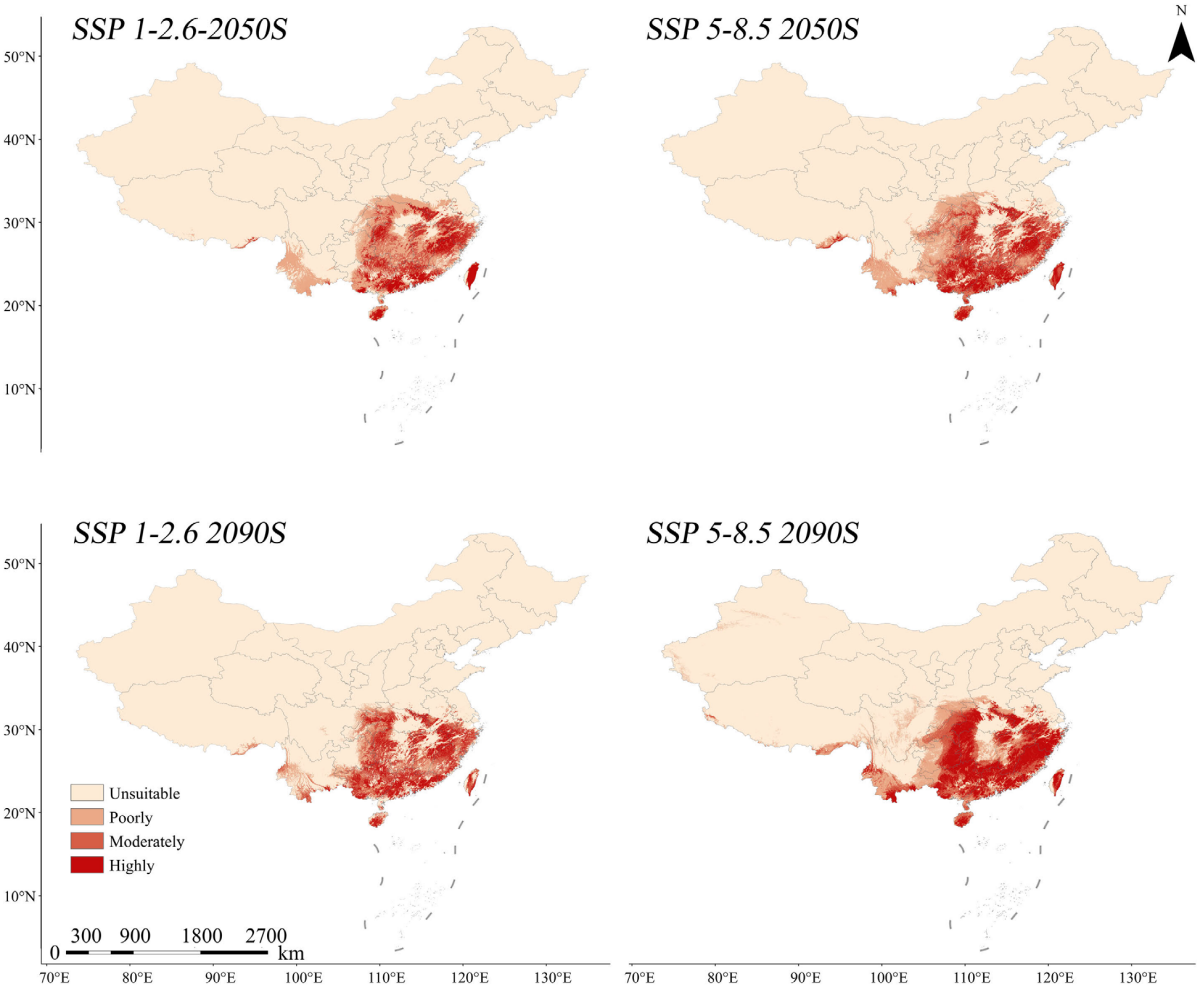
Models for two scenarios in two future periods (SSP 1‐2.6 and SSP 5‐8.5).

Under two future scenarios (SSP1‐2.6 and SSP5‐8.5), the contraction of the overall suitable habitat area for *S. incognitus* exhibits different patterns compared with the current Scenario 1, but generally demonstrates a declining trend (Table 3). In the SSP1‐2.6 (Shared Socioeconomic Pathway) scenario, the overall suitable habitat shows a shrinking trend, decreasing by 54.63 × 10^4^ km^2^ in the 2050s, approximately a 29.65% reduction in area; by the 2090s, it further shrinks by 73.71 × 10^4^ km^2^, approximately a 40.01% reduction in area. This suggests that the species' viability is diminishing under the green sustainability scenario, and based on this trend, the species' population is likely to decline, potentially leading to extinction. Under the SSP5‐8.5 scenario, the overall suitable habitat shrinks by 21.72 × 10^4^ km^2^ in the 2050s, a 21.07% reduction. However, by the 2090s, it increases by 17.09 × 10^4^ km^2^ compared to the 2050s, primarily in the highly suitable area, reducing the shrinkage from 24.2% in the 2050s to only 9.28% (Table 6). This suggests that, despite a large reduction in habitat under the fossil‐fueled development pathway, the species may return to the current scenario's habitat size in the 22nd century based on a gradual recovery of habitat and a gradual fragmentation of already fragmented habitats (Figure 6).

**TABLE 6:** Future scenario suitable habitat area statistical table.

Period	Current scenario 1	Current scenario 2	2050s		2090s	
Climate scenario	—	—	SSP1‐2.6	SSP5‐8.5	SSP1‐2.6	SSP5‐8.5
Total area of suitable habitat (×10^4^ km^2^)	184.24	140.11	129.61	145.42	110.53	167.15
Area of changes (×10^4^km^2^)						
Compared with the current scenario 1	—	—	−54.63	−38.82	−73.71	−17.09
Compared with the current scenario 2	—	—	−10.5	+5.31	−29.58	+27.04
Highly (×10^4^ km^2^)	61.95	37.19	36.82	40.64	36.34	66.16
Moderately (×10^4^ km^2^)	69.94	30.62	17.34	39.04	37.3	40.25
Poorly (×10^4^ km^2^)	52.35	72.3	75.45	65.74	36.89	60.74

*Note:* Includes the differences in the area of different‐level suitable habitats for Scenario 1, Scenario 2, and future scenarios (SSP 1‐2.6 and SSP 5‐8.5).

In comparison with the current Scenario 2, there is an expansion trend in the overall suitable habitat area for *S. incognitus* . Under the SSP1‐2.6 scenario, the suitable habitat shows an overall reduction trend, decreasing by only 10.5 × 10^4^ km^2^ in the 2050s; by the 2090s, it reduces by 29.58 × 10^4^ km^2^, a reduction in approximately 21.11%. In the SSP5‐8.5 scenario, suitable habitat expanded by 5.31 × 10^4^ km^2^ in the 2050s, a 3.79% increase. In the 2090s, it expands by 27.04 × 10^4^ km^2^, indicating a 19.30% increase compared with the current scenario. These results demonstrate that human activities significantly impact the suitable habitat of *S. incognitus* , with future habitat reductions being closely tied to anthropogenic disturbance.

### 3.6 Changes in Habitat Centroid

**FIGURE 7:**
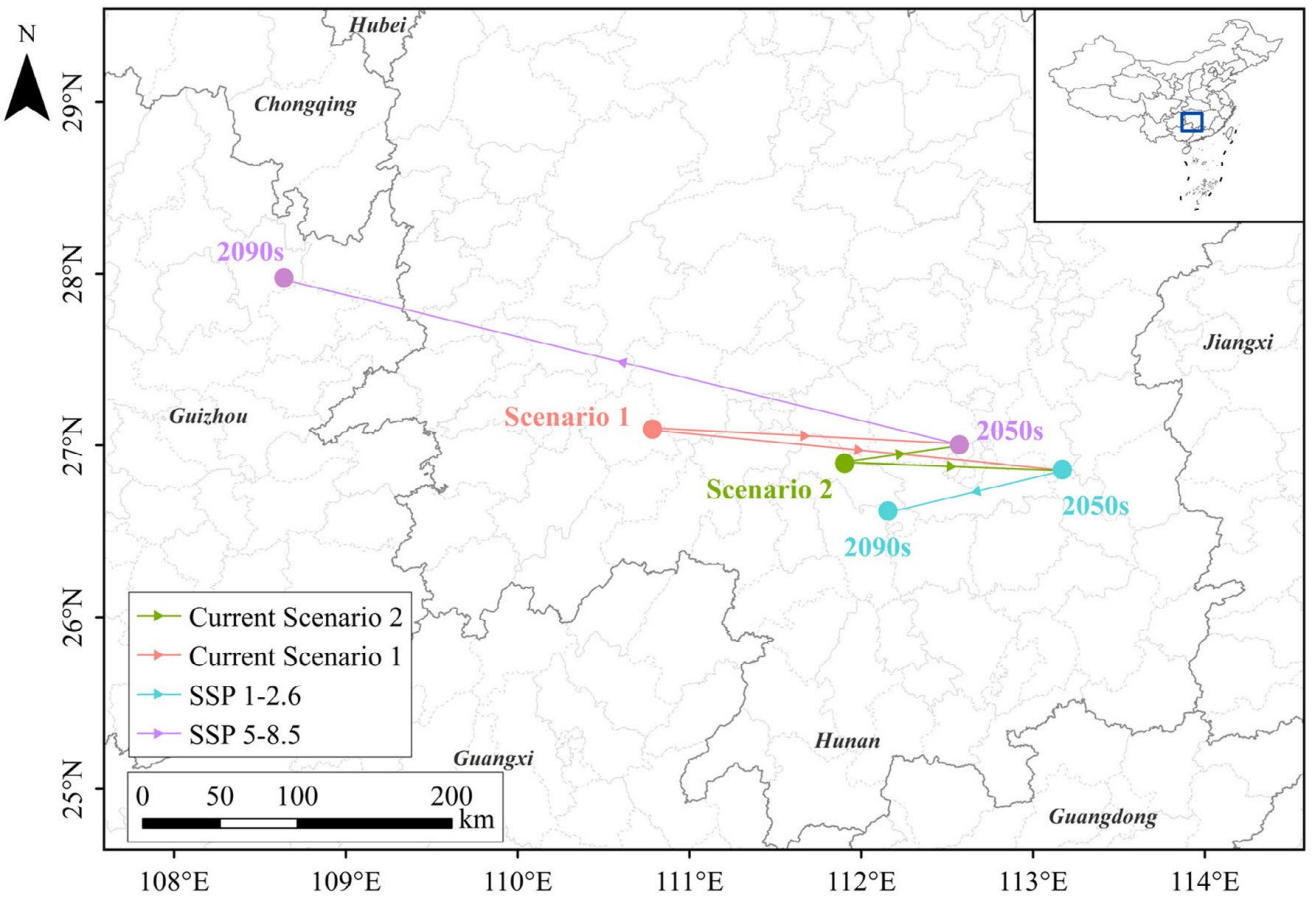
Centroid displacement plots under future climate conditions.

This study calculated the centroid variation of the overall suitable habitat for *S. incognitus* under different scenarios (Figure 7). In various scenarios, the distribution centroid of *S. incognitus* exhibits different trends. The results indicate that under current climate conditions, the centroid of *S. incognitus* ' suitable habitat in the natural environment scenario is located within Dongkou City, Hunan Province. Due to human activities, the centroid shifts southeastward by 113 km to Qidong County. Under future climate conditions, compared with the current Scenario 1, the centroid of the suitable habitat under the SSP1‐2.6 scenario moves southeastward by 238 km to Hengdong County in the 2050s and southwestward by 104 km to Qidong County in the 2090s. In this scenario, this migration pattern indicates a gradual shift of the centroid toward lower latitudes. In the SSP5‐8.5 scenario, the centroid moves southeastward by 177 km to Shigu District in the 2050s and northwestward by 403 km to Yinjiang County, Guizhou Province, in the 2090s. In this scenario, this shift reflects a gradual movement of the centroid toward higher latitudes. The centroid of *S. incognitus* ' suitable habitat exhibits two distinct movement patterns in the two future climate scenarios. Under the SSP1‐2.6 scenario, the centroid migrates toward lower latitudes, leading to a gradual reduction in the suitable habitat. Under the SSP5‐8.5 scenario, the centroid migrates toward higher latitudes, resulting in a gradual recovery of the suitable habitat. The comparative results indicate that, compared to the impact of human activities, the overall centroid migration aligns more closely with the natural environmental state. Thus, the results suggest that human activities have a limited impact on affect the migration of the centroid of the suitable habitat, with the overall centroid movement being more strongly influenced by natural environmental factors.

## 4 Discussion

### 4.1 Model Selection for Optimal Model

SDMs are widely used to address issues related to biodiversity conservation and environmental changes (Franklin 2023). However, these models may be subject to various conditions that could lead to decreased accuracy (Lawer 2024). Examples include the prediction of rare animals and poorer transferability (Hallgren et al. 2019; Mondanaro et al. 2023). Previous studies have indicated that ensemble models, which combine multiple models, often outperform single models in terms of predictive accuracy (Araújo and New 2007). In this study, we established an ensemble model by integrating individual models, which demonstrated better accuracy and effectively predicted the suitable habitat for *S. incognitus* . In addition, although the average performance of the independent model with the optimal TSS value may be superior, the actual results show that the independent model using the optimal AUC value performs better than the ensemble model obtained with the independent model using the optimal TSS value, which may be because training of the ensemble model requires combining models with varying levels of accuracy to improve the ensemble's overall performance. We designed two scenarios under current climatic conditions to predict the habitat of *S. incognitus* and analyzed the model simulation results.

Our findings indicate that including human activity factors in the model has a positive impact on predicting species habitat, as it significantly eliminates some suitable areas simulated by Scenario 1 that are located within urban areas. Although there is currently no direct evidence proving that *S. incognitus* cannot survive in urban environments, most studies suggest that this species is more likely to be fund in forests, shrubs, and meadows far from urban areas (Chen et al. 2017; Tang and Huang 2014).

### 4.2 Impacts of Environmental Factors and Human Activities on the Habitat of the *S. incognitus*

The integrated model's comprehensive output indicates that climatic factors, particularly precipitation, are the primary environmental determinants influencing the distribution of *S. incognitus* , while the importance of topographical factors and human activity is lower. This aligns with existing research demonstrating the significance of climate factors in shaping species distribution. For example, Thapa et al. (2018) revealed that annual precipitation and temperature are major contributors to the decline in the red panda ( *Ailurus fulgens* ) population, and Zhao et al. (2023) found that precipitation, temperature, and altitude are the main influencing factors for the habitat of Cabot's Tragopan ( *Tragopan caboti* ).

Although human activity factors occupy a relatively low proportion in the variable importance output of our model, their impact on habitat changes is significant (Pereira et al. 2010). We designed a randomized experiment to demonstrate that human activity factors are the sole cause of habitat changes, indirectly confirming the reliability of the model. A reevaluation of the Pearson correlation between the natural environmental factors and human activity factors used in the model indicates that human activity factors do not exhibit a high correlation with natural environmental factors.

**FIGURE 8:**
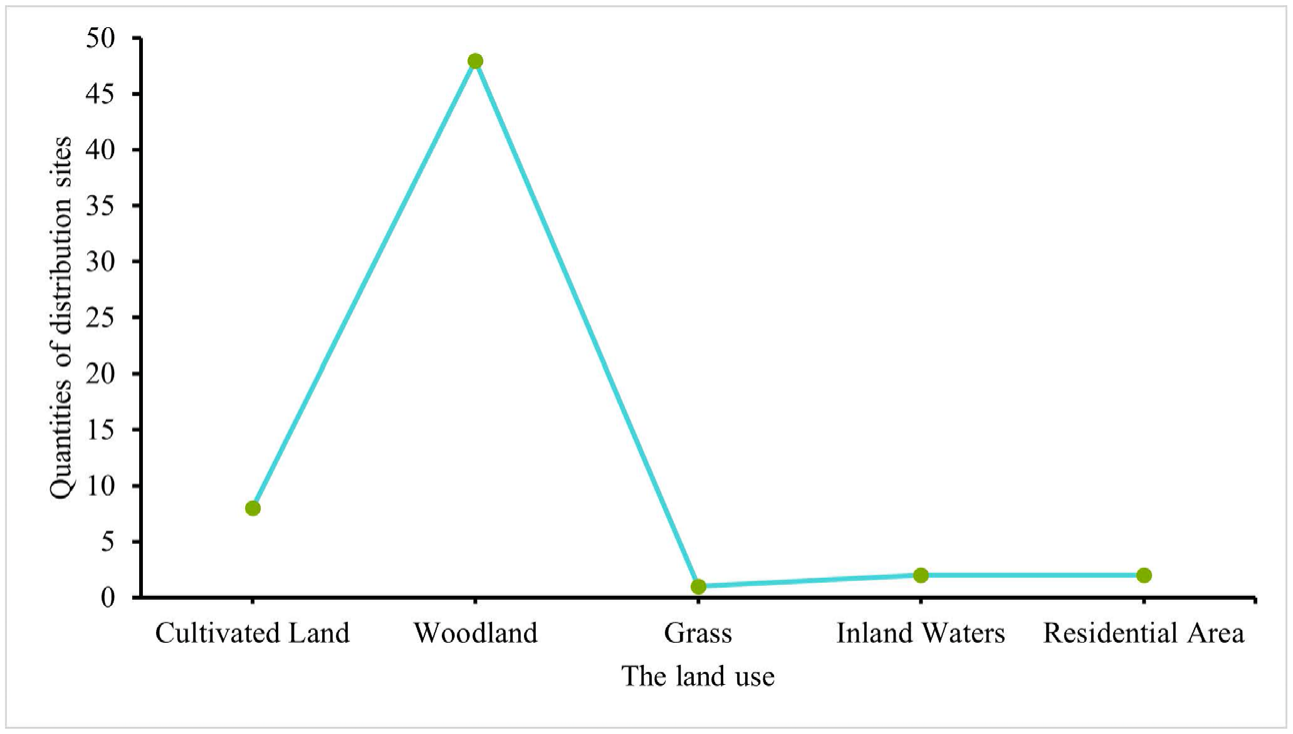
Attribute line chart for distribution points.

When comparing the importance results of all environmental variables, we found that the inclusion of human activity factors significantly decreased the importance of bio12, alt, and bio15. However, upon statistical assessment of the attributes corresponding to the patches of human activity factors, it was discovered that over 78% of the distribution sites are located in Woodland (Figure 8). This may explain the influence of this factor on the suitable habitat area of *S. incognitus*.

Habitat serves as the living space for animals, and the quality of the habitat environment significantly affects their survival and reproduction. Our study indicates that *S. incognitus* thrives in habitats such as forests and shrublands but is less prevalent in areas significantly impacted by human activities, such as cultivated land and urban areas. Wildlife habitats are increasingly influenced by human activities (Gaynor et al. 2018; Xi et al. 2020; Zhang et al. 2023). Studies suggesting that human development often accompanies habitat destruction, intensifying the risk of species decline (Li et al. 2024). Livestock farming significantly affects the food sources of wild animals, leading to the loss of their natural habitats (Li et al. 2017). Additionally, road construction impacts animal survival, prompting them to deliberately avoid their original habitats (Xu et al. 2021). The inclusion of human activity factors in our model revealed that the areas of suitable habitats for *S. incognitus* in the study area have reduced by 23.95%, signifying a significant adverse impact of human disturbances on habitat.

### 4.3 Changes in the Suitable Habitat of the *S. incognitus* in the Future

In future climate scenarios, the suitable habitat of *S. incognitus* exhibits a noticeable contraction compared with the current climate. However, two distinct scenarios (SSP1‐2.6 and SSP5‐8.5) reveal different centroid migration trends. In the SSP1‐2.6 scenario, the habitat gradually moves southward and diminishes progressively. Conversely, in the SSP5‐8.5 scenario, the centroid experiences a southward shift, followed by a sudden northward movement, leading to a gradual expansion after an initial reduction in habitat area.

The current importance of climate variables indicates that precipitation is the most critical factor influencing the distribution of *S. incognitus* . Existing research has shown that increasing CO_2_ concentrations, coupled with the joint impact of temperature and precipitation changes, lead to significant horizontal shifts and range contractions in Chinese climate regions (Zheng, Yin, and Li 2010; Hu, Xiao, and Li 2020). Different concentrations of greenhouse gas emissions have led to two distinct distribution trends for *S. incognitus* habitats.

In the SSP1‐2.6 scenario, as the climate warms and precipitation increases, the overall reduction in *S. incognitus* habitat from 2050 to 2090 tends to stabilize due to the cessation of greenhouse gas emissions around 2050 (Hurtt et al. 2020). In the SSP5‐8.5 scenario, extreme human greenhouse gas emissions lead to a drastic rise in temperature (Jiang et al. 2020), intensifying the northward shift of the precipitation belt and increasing overall precipitation. As a result, the suitable habitat for *S. incognitus* expands to higher latitudes, gradually restoring the previously fragmented habitat due to the change in climate conditions.

Numerous studies suggest that under future climate conditions, species' habitats will gradually shrink (Tian 2021; Rezaei et al. 2023; Lawer 2024). These studies collectively emphasize the significant impact of climate change on species' habitats, regardless of the animal species (Hooper et al. 2012; Urban 2015; Shivanna 2022).

The future climate factors utilized are from CMIP6, which are derived from the collaborative efforts of six Integrated Assessment Models (IAM) based on Shared Socioeconomic Pathways (SSPs) scenarios (Bosello and Cian 2014; Calvin et al. 2017; Fricko et al. 2017; Kriegler et al. 2017; Fujimori et al. 2017; Van Vuuren et al. 2017). The SSPs incorporate future land‐use data (Jiang, Wang, et al. 2020), making the comparison of results under anthropogenic disturbance conditions with future scenarios more reasonable.

**FIGURE 9:**
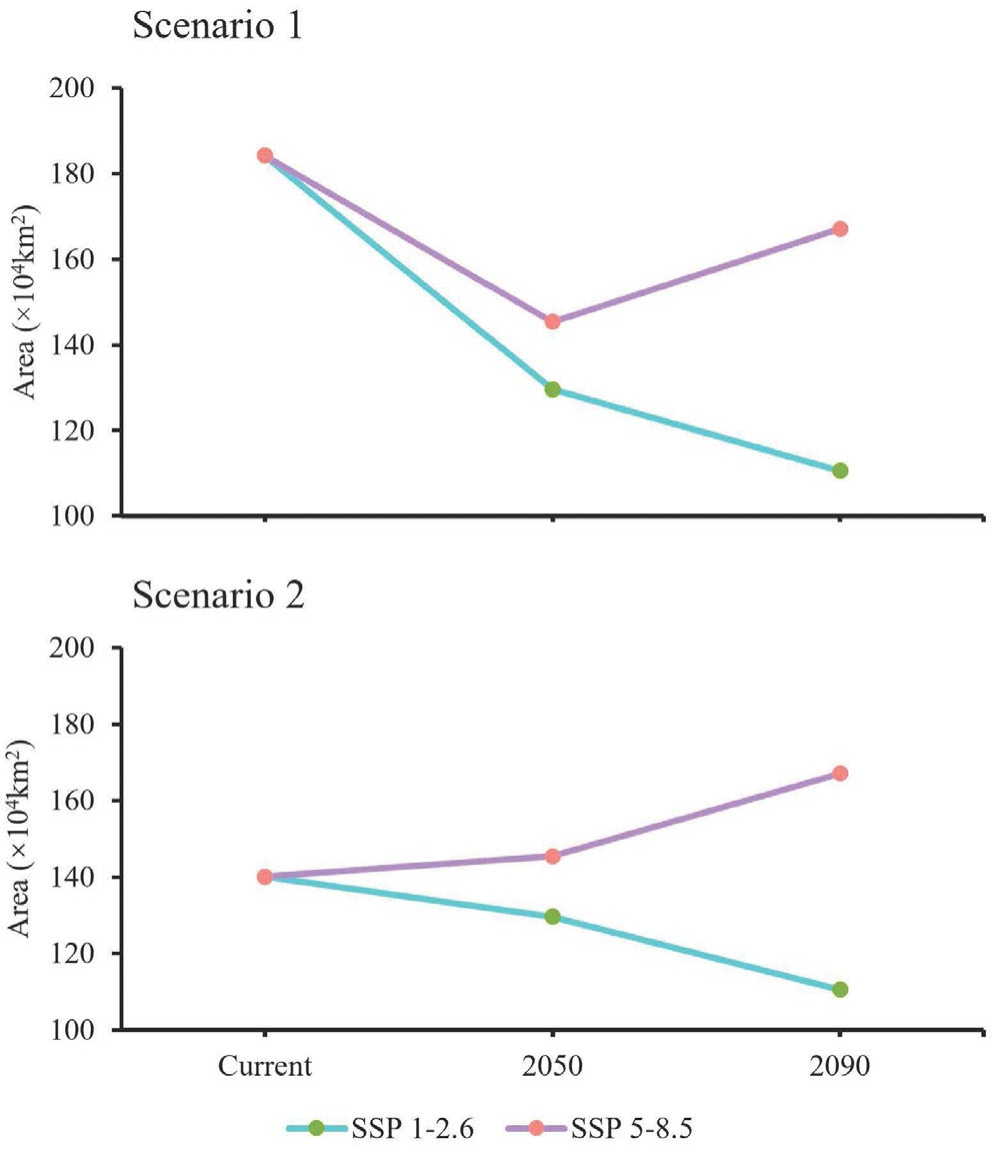
Trends in area under different scenarios.

Under the SSP1‐2.6 and SSP5‐8.5 scenarios, *S. incognitus* exhibits different expansion and contraction trends (Figure 9). The expansion trend is more pronounced in Scenario 2 than in Scenario 1, which illustrates the impact of human activity on animal habitats. Furthermore, this is supported by the varying concentrations of greenhouse gas emissions in different future scenarios, which lead to different increases in precipitation (Jiang, Zhou, et al. 2020; Li et al. 2022). This underscores the importance of precipitation as the most critical factor influencing the suitable habitat of *S. incognitus*. Therefore, it may be that human activities are gradually affecting the Earth's land and climate. Further studies in this direction should be conducted to validate additional climate scenarios. Human activity should not be ignored when predicting species' habitats, especially when using CMIP6 as a future factor for expansion/contraction analysis. The impacts of climate change caused by increasing anthropogenic emissions of greenhouse gases on human society and natural systems are indisputable (Jiang, Zhou, et al. 2020).

## 5 CONCLUSION

In this study, we utilized the Biomod2 platform to simulate the distribution of *S. incognitus* under current and future climate scenarios, considering both natural and anthropogenic factors. Our results demonstrate that anthropogenic disturbances have a significant negative impact on the suitable habitat of *S. incognitus* , leading to a reduction of 44.13 × 10^4^ km^2^ under current climate conditions compared with natural scenarios. Furthermore, we identified precipitation as the most crucial environmental factor influencing the potential distribution of this species. Future habitat predictions for *S. incognitus* revealed different patterns of expansion and contraction under different scenarios, with human activities remaining the primary influence. This finding emphasizes the substantial role of anthropogenic factors in altering the environment and affecting wildlife habitats.

Based on our findings, we recommend the implementation of targeted conservation measures to protect the habitat of *S. incognitus* and other wildlife species. These measures should include progressively reducing human impact on the environment to protect nature. Additionally, future research should incorporate a broader range of socioeconomic data to better understand the complex interplay between human activities and species' habitats. Our study highlights the importance of considering both anthropogenic and climatic factors when assessing species' distributions and developing conservation strategies. Adopting a comprehensive approach will enhance biodiversity protection amidst growing human pressures and global environmental change.

## Author Contributions

**Kai Chen:** data curation (equal), formal analysis (equal), methodology (equal), software (equal), visualization (equal), writing – original draft (equal). **Li Ma:** data curation (equal), formal analysis (equal), funding acquisition (lead), methodology (equal), project administration (equal), software (supporting), supervision (lead), validation (equal), writing – original draft (lead), writing – review and editing (lead). **Weijun Jiang:** formal analysis (equal), visualization (equal). **Lijin Wang:** investigation (equal), writing – original draft (equal), writing – review and editing (equal). **Li Wei:** investigation (equal), writing – original draft (equal), writing – review and editing (equal). **Hongji Zhang:** data curation (equal), formal analysis (equal), resources (equal). **Ruhao Yang:** data curation (equal), formal analysis (equal), resources (equal).

## Conflicts of Interest

The authors declare no conflicts of interest.

## Supporting information


Data S1.

## Data Availability

The datasets generated and/or analyzed during the current study are available in the supplemental text (Table S1). The distribution data and sources of *Sphenomorphus incognitus* are shown in S1. The climate data in this study were accessed through the Worldclim database (http://www.worldclim.org/) (accessed on April 6, 2023). The future climate data represented long‐term average climatic conditions in 2050s (average for 2041–2060) and 2090s (average for 2081–2100), as modeled by CMIP6 using the CCSM4 climatic system (http://www.worldclim.org/). The topographic data (elevation slope aspect and topographic shadow) were derived from digital elevation model (DEM) (https://www.gebco.net/). The human data (the land use and cover change) were from resource and environmental science data platform (https://www.resdc.cn).
