# Supplementary material for: Anthropogenic Disturbance and Climate Change Impacts on the Suitable Habitat of Sphenomorphus incognitus in China
Source: Ecol Evol. 2025 Jan 18;15(1):e70848. doi: 10.1002/ece3.70848 (PMC11748457; doi:10.1002/ece3.70848)
Supplement: Supplementary file 1 — Data S1. [file ECE3-15-e70848-s001.docx]

**Supplemental Text**

**TABLE S1** The distribution data and sources of *Sphenomorphus incognitus.*

| **Num** | **Long** | **Lat** | **Resource** |
| --- | --- | --- | --- |
| 1 | 109.805078 | 25.92420748 | Li et al., 2023 |
| 2 | 109.5631781 | 26.30641255 | Li et al., 2023 |
| 3 | 109.9779275 | 26.31022186 | Li et al., 2023 |
| 4 | 117.7712511 | 27.93977496 | Cheng et al., 2023 |
| 5 | 112.8310764 | 24.27910667 | Huang, 2023 |
| 6 | 109.9032441 | 18.66985872 | Li et al., 2022 |
| 7 | 110.0141375 | 18.3873336 | Li et al., 2022 |
| 8 | 109.5755622 | 19.01877527 | Li et al., 2022 |
| 9 | 109.1187324 | 19.09042915 | Li et al., 2022 |
| 10 | 118.8750247 | 32.07453343 | Lin, 2022 |
| 11 | 113.4489945 | 22.46834518 | Hua et al., 2021 |
| 12 | 113.1294443 | 24.51147767 | Zheng et al., 2021 |
| 13 | 110.6285504 | 25.55053421 | Liu, 2021 |
| 14 | 112.7411521 | 25.70684188 | Lei, 2021 |
| 15 | 110.0175241 | 31.47709841 | Xing et al., 2021 |
| 16 | 115.0930858 | 23.78142225 | He et al., 2020 |
| 17 | 113.1986671 | 24.79035377 | Yang et al., 2020 |
| 18 | 118.1518999 | 29.45617868 | Li & Wu, 2019 |
| 19 | 114.5335242 | 22.50688538 | Zhang et al., 2019 |
| 20 | 113.3538011 | 23.15687174 | Luo, 2018 |
| 21 | 113.86828 | 23.63966741 | Luo, 2018 |
| 22 | 113.74547 | 23.73012344 | Ma, 2018 |
| 23 | 113.9829537 | 31.67022997 | Chen, 2018 |
| 24 | 114.4434478 | 25.08201704 | Yang, 2018 |
| 25 | 118.8453511 | 28.3916165 | Chen et al., 2017 |
| 26 | 118.968903 | 28.75941802 | Chen et al., 2017 |
| 27 | 117.1971861 | 27.80079976 | Chen et al., 2017 |
| 28 | 117.3847053 | 27.95354609 | Chen et al., 2017 |
| 29 | 117.8861959 | 28.00637531 | Chen et al., 2017 |
| 30 | 118.2737191 | 28.14079298 | Chen et al., 2017 |
| 31 | 109.6449312 | 32.07029536 | Tian, 2017 |
| 32 | 110.0454051 | 31.7006726 | Tian, 2017 |
| 33 | 110.6982291 | 31.88615025 | Tian, 2017 |
| 34 | 118.1179506 | 26.64166672 | Gong, 2017 |
| 35 | 114.0564326 | 23.27058039 | Li, 2015 |
| 36 | 114.1978101 | 22.59061634 | Tang et al., 2015 |
| 37 | 108.0546052 | 25.15397209 | Wang, 2014 |
| 38 | 117.4366265 | 26.69212513 | Wang et al., 2013 |
| 39 | 114.2629911 | 24.73670503 | Rao et al., 2013 |
| 40 | 110.2001111 | 24.13110492 | Zeng, 2012 |
| 41 | 109.3416141 | 22.1799568 | Zeng, 2012 |
| 42 | 107.458587 | 21.91594725 | Zeng, 2012 |
| 43 | 112.5398093 | 23.1596706 | Gong et al., 2012 |
| 44 | 114.9112201 | 29.93332909 | Dai et al., 2011 |
| 45 | 114.5087171 | 29.41181564 | Dai et al., 2011 |
| 46 | 113.8349649 | 29.18078205 | Dai et al., 2011 |
| 47 | 114.6351698 | 31.55037039 | Dai et al., 2011 |
| 48 | 115.6323588 | 30.99356727 | Dai et al., 2011 |
| 49 | 111.01643 | 26.41630209 | Guo et al., 2007 |
| 50 | 113.9240921 | 31.85153382 | Deng et al., 2006 |
| 51 | 110.5381143 | 32.97212781 | Yu et al., 2005 |
| 52 | 110.4788053 | 29.22936753 | Yu et al., 2005 |
| 53 | 110.3857997 | 29.20819616 | Yu et al., 2005 |
| 54 | 101.0209715 | 24.70777295 | He et al., 2002 |
| 55 | 114.1527665 | 22.39519767 | Farm & Garden, 2005 |
| 56 | 121.5518117 | 22.05861857 | Huang, 2000 |
| 57 | 120.7741776 | 22.00108879 | Zhao et al., 1999 |
| 58 | 98.30978394 | 24.91134942 | Zhao et al., 1999 |
| 59 | 114.2709875 | 22.63562705 | https://finance.sina.com.cn/jjxw/2022-05-20/doc-imcwiwst8557069.shtml |
| 60 | 118.936395 | 30.08548754 | https://baijiahao.baidu.com/s?id=1741852185751084921&wfr=spider&for=pc |
| 61 | 121.1560518 | 29.46654989 | This Study |
| 62 | 117.6251109 | 27.66853113 | This Study |
| 63 | 117.8554574 | 27.87415961 | This Study |
| 64 | 117.7084345 | 27.72625753 | This Study |
| 65 | 117.7980324 | 27.62949364 | This Study |
| 66 | 117.9625296 | 27.67046027 | This Study |
| 67 | 117.737092 | 27.69099687 | This Study |
| 68 | 117.7490531 | 27.70742946 | This Study |
| 69 | 117.7731045 | 27.70075105 | This Study |
| 70 | 117.641512 | 27.70042549 | This Study |
| 71 | 117.869528 | 27.85318053 | This Study |
| 72 | 117.8166199 | 27.69764007 | This Study |
| 73 | 117.9526211 | 27.67379833 | This Study |
| 74 | 117.6489364 | 27.67933006 | This Study |
| 75 | 117.6596045 | 27.65734006 | This Study |
| 76 | 117.648103 | 27.653749 | This Study |
| 77 | 117.643943 | 27.63886189 | This Study |
| 78 | 117.643782 | 27.64325355 | This Study |
| 79 | 117.6431177 | 27.64622964 | This Study |
| 80 | 117.4826397 | 27.65006382 | This Study |
| 81 | 117.6752718 | 27.7747575 | This Study |
| 82 | 117.762067 | 27.66136783 | This Study |

**FIGURE S1** Random pairing experiment model figure. Random experimental design for two scenarios in the current climate, including paired group numbers and model rank numbers extracted using Rand Between.

**
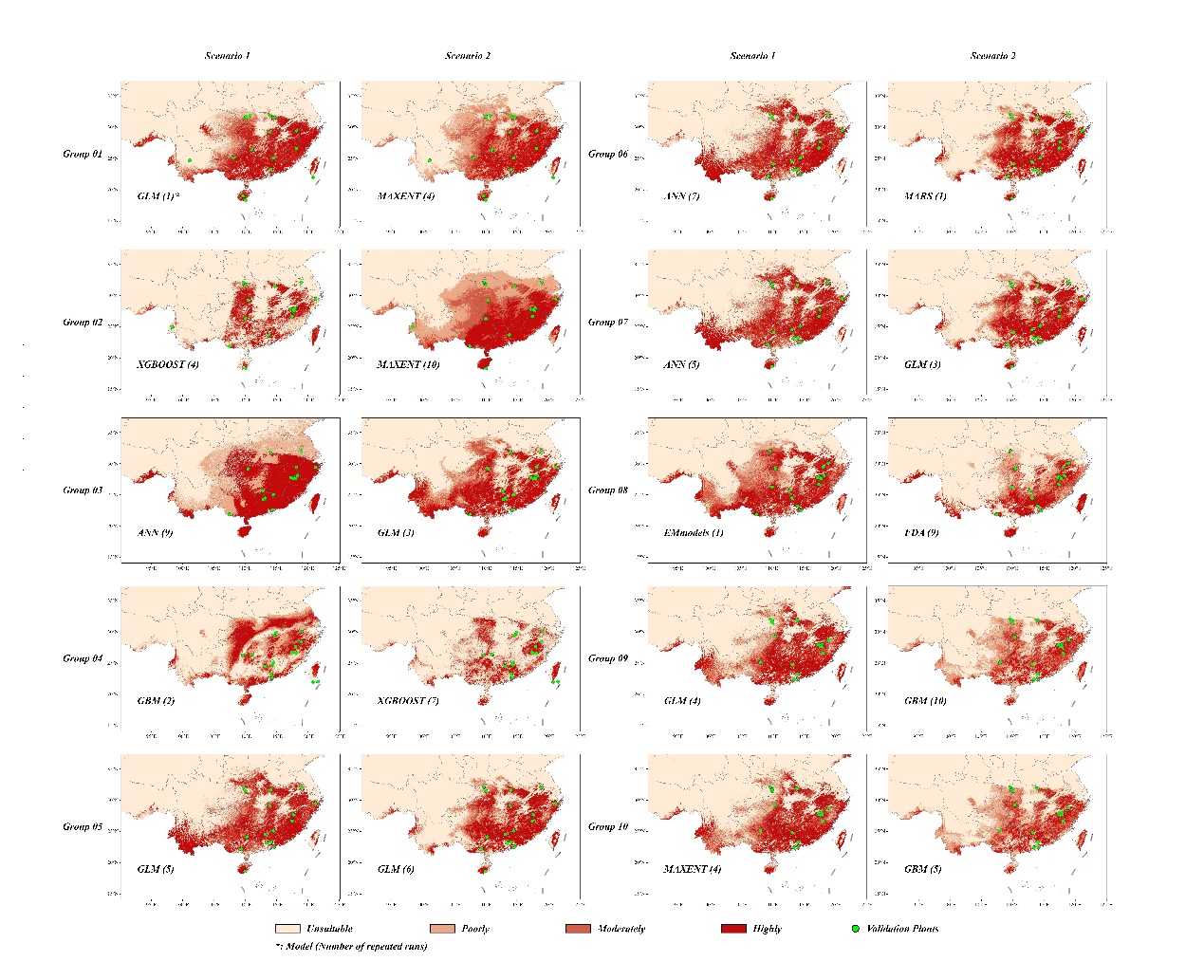
**
